# The efficacy of the treat-repair-treat strategy for severe pulmonary arterial hypertension associated with congenital heart disease: a meta-analysis

**DOI:** 10.1186/s12872-023-03606-z

**Published:** 2023-11-20

**Authors:** Zhiyuan Wang, Xiaobing Li, Mengxuan Li, Jun Peng, Huijun Zhang

**Affiliations:** Department of Cardiac Surgery, The First Hospital of Hebei Medical University, No. 89, Donggang Road, Yuhua District, Shijiazhuang City, Hebei Province China

**Keywords:** Pulmonary arterial hypertension, Congenital heart disease, Treat-repair-treat, Targeted drugs

## Abstract

**Background:**

This meta-analysis was conducted to evaluate the efficacy of the treat-repair-treat (TRT) strategy in the treatment of severe pulmonary arterial hypertension with congenital heart disease (PAH-CHD).

**Methods:**

PubMed, EMBASE, Cochrane and Web of Science online databases were searched by two independent investigators for studies that used the TRT strategy for PAH-CHD, and the retrieved studies were reviewed by a third investigator. The main outcomes were pulmonary artery pressure (PAP), pulmonary vascular resistance (PVR), 6-minute walk distance (6MWD), and transcutaneous oxygen saturation (SpO_2_). The changes were compared between follow-up and baseline. Stata version 14.0 was used for data analysis. A random-effects model was selected for meta-analysis. Subgroup analysis and meta-regression were used to find the source of heterogeneity.

**Results:**

A total of 335 patients from 9 single-arm studies were included. Meta-analysis showed significant reductions in PAP and PVR and improvements in 6MWD and SpO_2_ (PAP: SMD -2.73 95% CI -2.97, − 2.50 *p* = < 0.001; PVR: SMD -1.27 95% CI -1.53, − 1.02 *p* = < 0.001; 6MWD: SMD 1.88 95% CI 1.49, 2.27 *p* = < 0.001; SpO_2_: SMD 3.72 95% CI 3.13, 4.32 *p* = < 0.001). Subgroup analysis showed that younger patients had better efficacy, and the change in SpO_2_ was an indication for patient selection. The combined mortality rate was 5% at follow-up.

**Conclusions:**

In this meta-analysis, we demonstrated that the TRT strategy may have positive effects on haemodynamics and cardiac function in patients with severe PAH-CHD at short-term follow-up. Our analysis suggests that changes in age and SpO_2_ may be related to patient prognosis.

**Trial registration:**

The protocol was registered on the PROSPERO website with the registration number CRD42022366552. The relevant registration information can be obtained from the website https://www.crd.york.ac.uk/prospero/#searchadvanced.

## Background

Pulmonary arterial hypertension associated with congenital heart disease (PAH-CHD) is a serious cardiovascular disease. The left-to-right shunt results in increased blood flow and pressure in the pulmonary vasculature [1]. Persistent exposure of the pulmonary vasculature to increased blood flow, as well as increased pressure, may result in pulmonary obstructive arteriopathy [2]. Surgical repair at an early stage can correct the left-to-right shunt and improve patient prognosis. In the advanced stages of the disease, the pulmonary artery is irreversibly diseased, and the patient will lose the opportunity for surgery. Patients experience reduced exercise tolerance, heart failure [3], and even death. Targeted drugs, which have been invented in the last 20 years, include endothelin receptor antagonists (ERAs), phosphodiesterase type-5 inhibitors (PDE5is), prostacyclin (PC), and soluble guanylate cyclase stimulators (sGC). Combined drug therapy can improve patients’ exercise capacity and reduce pulmonary vascular resistance (PVR) [4]. In some cases, patients with severe PAH-CHD have successfully completed surgery after receiving targeted drug therapy [5–7]. In 2010, a large case series from China suggested that patients who are sensitive to targeted drug therapy have access to surgery [8]. The development of targeted drugs has brought new hope for patients [8–16]. It has been suggested that endothelin receptor antagonists may have antiproliferative effects causing reverse remodelling in the pulmonary circulation [17]. This concept has made surgical treatment possible. The preoperative and postoperative administration of targeted drugs to enable patients with severe PAH-CHD to complete surgery for congenital heart disease is called the TRT strategy, and the treatment guidelines have changed as a result. According to the 2022 ESC/ERS Guidelines for the diagnosis and treatment of PAH [18], in patients with ASD and a PVR > 5 Wu that declines to < 5 Wu with PAH treatment, shunt closure may be considered. In patients with ASD and a PVR > 5 WU despite PAH treatment, shunt closure is not recommended. However, there are many questions about the efficacy of the TRT strategy. First, pulmonary hypertension crisis is an important cause of perioperative death in patients with CHD [19, 20]. Second, many patients continue to have PAH after surgery. In Takaya et al. [9] report, 69% of patients were still taking targeted drugs at follow-up, and 19% were increasing their drug use. Therefore, the purpose of this study was to collect relevant clinical studies for meta-analysis to evaluate the efficacy of the TRT strategy for patients with PAH-CHD. We summarized the prognostic factors of patients, hoping to help doctors judge the feasibility of surgery.

## Methods

This study was conducted in adherence with the Preferred Reporting Items for Systematic Reviews and Meta-Analyses (PRISMA) statement [21]. Three reviewers independently conducted the literature search, screening evaluation, and data extraction.

### Retrieval strategy

The PubMed, Embase, Cochrane and Web of Science online databases were searched for relevant studies by three reviewers. The following keyword combinations were used to retrieve relevant studies: (“Treat Repair” or “Treat Close” or “specific medications” or “targeted drugs” or “specific drugs” or “targeted medications”) and (“pulmonary arterial hypertension associated with congenital heart disease” or “PAH-CHD” or “CHD-PAH”). Included were articles published in all languages, studies carried out on human subjects and articles published from the establishment of the database to November 1, 2022. The search strategies were adapted according to the characteristics of the databases. The reference lists of the included articles were manually retrieved to identify additional relevant studies.

### Inclusion and exclusion criteria

Studies that met the following criteria were included: (1) population: patients with PAH associated with congenital heart disease and a mean PVR > 5 Wu or a mean mPAP> 45 mmHg without treatment; (2) intervention: interventional occlusion or surgical repair after treatment with PAH-specific medications; and (3) comparison: baseline data were compared with data at follow-up. The exclusion criteria were as follows: (1) overviews, reviews, seminar papers, comments, reports, letters, animal experiments and duplicate publications were excluded; (2) lack of right cardiac catheterization data; and (3) PAH after surgery for congenital heart disease.

### Data extraction and quality assessment

The extracted items included (1) details of the articles (first author, publication year, sample size); (2) sample characteristics (age, sex, diagnosis of CHD); and (3) intervention measures (type of targeted drugs, duration of medication, mode of operation and follow-up duration). (4) Main outcomes: mean pulmonary artery pressure (mPAP) or systolic pulmonary artery pressure (sPAP), PVR, 6MWD, SpO_2,_ Qp:Qs and death toll. Baseline and last follow-up data were collected. According to the study of McGrath et al. [22], medians and interquartile ranges were converted into means and standard deviations. If the data were not given in the article and could not be obtained from the author, we used WebPlotDigitizer version 4.1 [23] to estimate the interquartile ranges based on box plots. We calculated the interquartile ranges for each article three times independently and chose the average as the final value for analysis.

The quality of the included studies was evaluated according to the MINORS (Methodological Index for Nonrandomized Studies) checklist [24]. This tool was specifically developed and validated to evaluate the quality of nonrandomized surgical studies. It includes 12 items; the last 4 are specific for comparative studies. The score for the items varies from 0 to 2 (0, not reported; 1, reported but poorly done or inadequate; 2, reported but well done and adequate). The ideal global score was 16 for a noncomparative study and 24 for a comparative study. This evaluation was performed by two independent reviewers who tried to reach a consensus in case of disagreement.

### Statistical analysis

Statistical software Stata 14.0 was used for meta-analysis. For continuous variables, the standardized mean difference (SMD) was used for calculations, and a fixed effects model was used for analysis. Heterogeneity was tested by Cochrane’s Q test and quantified by the I^2^ statistic. When the data had great heterogeneity (I^2^ > 50%), the random effects model was used for analysis. Subgroup analysis was used to compare the efficacy of different patients, and meta-regression was used to determine the source of heterogeneity. Publication bias was analysed by Egger’s bias tests. A *p* value< 0.05 was considered statistically significant (significant bias). The mortality rate was recorded, and the estimated effect (ES) for mortality estimates with the corresponding 95% CI was calculated.

## Results

### Characteristics of eligible studies and quality assessment

**Fig. 1:**
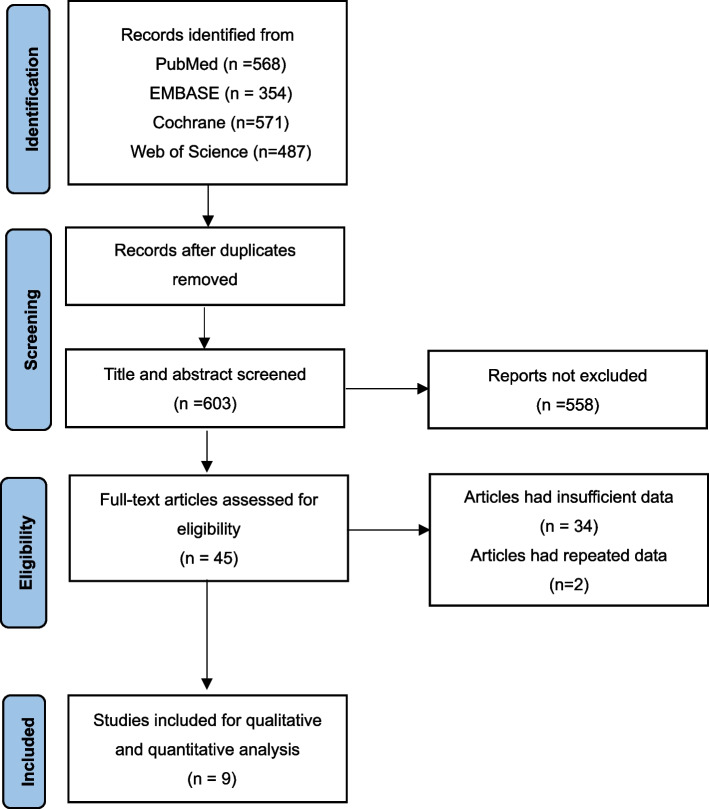
Flow diagram for retrieving eligible articles

The search retrieved 603 results. A total of 558 irrelevant articles were excluded. We reviewed the full texts of the remaining 45 articles and excluded 36 articles with incomplete and repeated data. Finally, we included 9 single-arm studies with 335 patients. The flow chart of the research selection process is shown in Fig. 1. One study involved eight patients with Eisenmenger syndrome. Because Eisenmenger syndrome is often accompanied by severe PAH and these patients are difficult to treat and have a very poor prognosis, we still included this study to avoid data loss and bias. Details of the articles and sample characteristics are collected in Table 1. Intervention measures and main outcomes are displayed in Table 2. The screening criteria and the number of deaths are shown in Table 3. We also collected the proportion of patients taking targeted drugs at the last follow-up in Table 3. Our meta-analysis included 9 studies with a MINOR score of 12-13 each, suggesting that low-quality studies were not included.

**Table 1:** Sample characteristics and intervention measures

Author	Year	N	Sex (m/f)	Age (year)	Diagnosis	Duration of medication(m)	Follow-up duration (m)	MINORS
Takaya et al. [9]	2021	42	10/32	51 ± 18	ASD	9	33	12
Thomaz et al. [10]	2019	33	12/18	0.8(median)	VSD\PDA\A-V canal	6	6	13
Huang et al. [11]	2011	38	–	3.04 ± 1.98	ASD\VSD\PDA	5-120 days	117.6	12
Hu et al. [12]	2015	31	19/21	29.19 ± 8.51	VSD	7.29	37.9	12
		8	3/5	22.75 ± 3.77	VSD and ES	12.38	31.50	12
Kijima et al. [13]	2015	8	0/8	37 ± 15	ASD	–	19	12
Bradley et al. [14]	2018	19	4/15	37 + 23	ASD	24	52.8	12
Liu et al. [15]	2014	56	25/31	17.2 ± 9.3	VSD	4.4	27.9	13
He et al. [16]	2020	14	1/13	27.9 ± 7.4	ASD	10.7	21.1	12
Liu et al. [8]	2010	86	51/35	2 ± 1.8	d-TGA\TBA	13.4 days	42.1	12

*m/f* male/female, *m* month, *ASD* atrial septal defect, *VSD* ventricular septal defect, *PDA* patent ductus arteriosus, *A-V canal* atrioventricular septal defect, *ES* Eisenmenger syndrome, *d-TGA* dextrotransposition of the great arteries, *TBA* Taussig-Bing anomaly

**Table 2:** Main outcomes

Author	Targeted drug	Mode of operation	mPAP or sPAP (mmHg)	PVR (Wu)	6MWD (m)	SpO_2_	Qp/Qs
Takaya et al. [9]	ERA\PDE5i\sGC\PC	intervention	A:78.00 ± 26.00 B:38.00 ± 10.00	A:6.90 ± 3.20 B:3.20 ± 1.70	- -	- -	A:1.90 ± 0.80 -
Thomaz et al. [10]	PDE5i	–	A:47.77 ± 14.77 B:25.27 ± 7.64	A:5.70 ± 3.69 B:2.97 ± 1.78	- -	- -	A:2.09 ± 1.02 B:2.43 ± 1.03
Huang et al. [11]	ERA\PDE5i\PC	surgery	A:70.5.0 ± 11.92 B:30.95 ± 10.92	A:20.51 ± 8.90 B:9.20 ± 3.63	- -	- -	- -
Hu et al. [12]	ERA\PDE5i\PC	valved patch repair	A:79.61 ± 6.88 B:< 50	- -	- -	- -	A:1.24 ± 0.21 -
	ERA\PDE5i\PC	valved patch repair	A:82.54 ± 6.49 B:59.05 ± 14.52	- -	- -	A:85.79 ± 2.29 B:91.13 ± 3.72	A:0.84 ± 0.13 -
Kijima et al. [13]	ERA\PDE5i\PC	intervention	A:104.00 ± 27.00 B:40.00 ± 9.00	- -	- -	- -	A:1.39 ± 0.41 -
Bradley et al. [14]	ERA\PDE5i\sGC\PC	–	A:48.00 ± 13.00 B:36.63 ± 12.76	A:7.50 ± 4.30 B:5.05 ± 2.88	A:366 ± 137 B:486 ± 89	- -	A:2.2 ± 1.5 B:1.11 ± 0.46
Liuet al [15].	ERA\PDE5i	surgery	A:67.90 ± 9.90 B:22.50 ± 6.40	- -	A:337.6 ± 23.3 B:424.6 ± 48.3	A:89.90 ± 2.10 B:97.30 ± 1.10	- -
He et al. [16]	ERA\PDE5i	12 cases: intervention 2 cases: surgery	A:57.10 ± 7.40 B:32.40 ± 14.10	A:8.70 ± 2.90 B:4.10 ± 2.70	- -	- -	A:1.40 ± 0.25 B:1.00 ± 0.00
Liu et al. [8]	ERA\PDE5i	surgery	A:64.90 ± 13.00 B:22.40 ± 8.70	- -	- -	- -	- -

A: Baseline; B: The last follow-up

**Table 3:** Patient screening and prognosis

Author	Screening of patients	Targeted drugs at the final follow-up	Deaths
Takaya et al. [9]	CCC:PVR < 5.0–7.0 Wu\Qp:Qs > 1.5 Left-to-right shunt	100%	1
Thomaz et al. [10]	VT: PVR < 6.0 Wu and PVR/SVR < 0.3	82%	3
Huang et al. [11]	SpO_2_ > 93% Left-to-right shunt	–	0
Hu et al. [12]	VT: Qp/Qs > 1.5 and PVR/SVR < 2/3 Left-to-right shunt	63%	2
Kijima et al. [13]	CCC:Qp/Qs ≥1.5 and PVR < 8 Wu	100%	0
Bradley et al. [14]	CCC:PVR < 6.4 Wu and 6MWD > 300 m	–	0
Liu et al. [15]	SpO_2_ > 93%	42%	0
He et al. [16]	VT:PVR < 5 Wu or Qp/Qs > 1.5	86%	0
Liu et al. [8]	increase of SpO_2_ > 5%	–	6

*CCC* conventional cardiac catheterization, *VT* vasodilation test, *Qp:Qs* pulmonary to systemic blood flow ratio, *SVR* systemic vascular resistance

### Meta-analysis

**Fig. 2:**
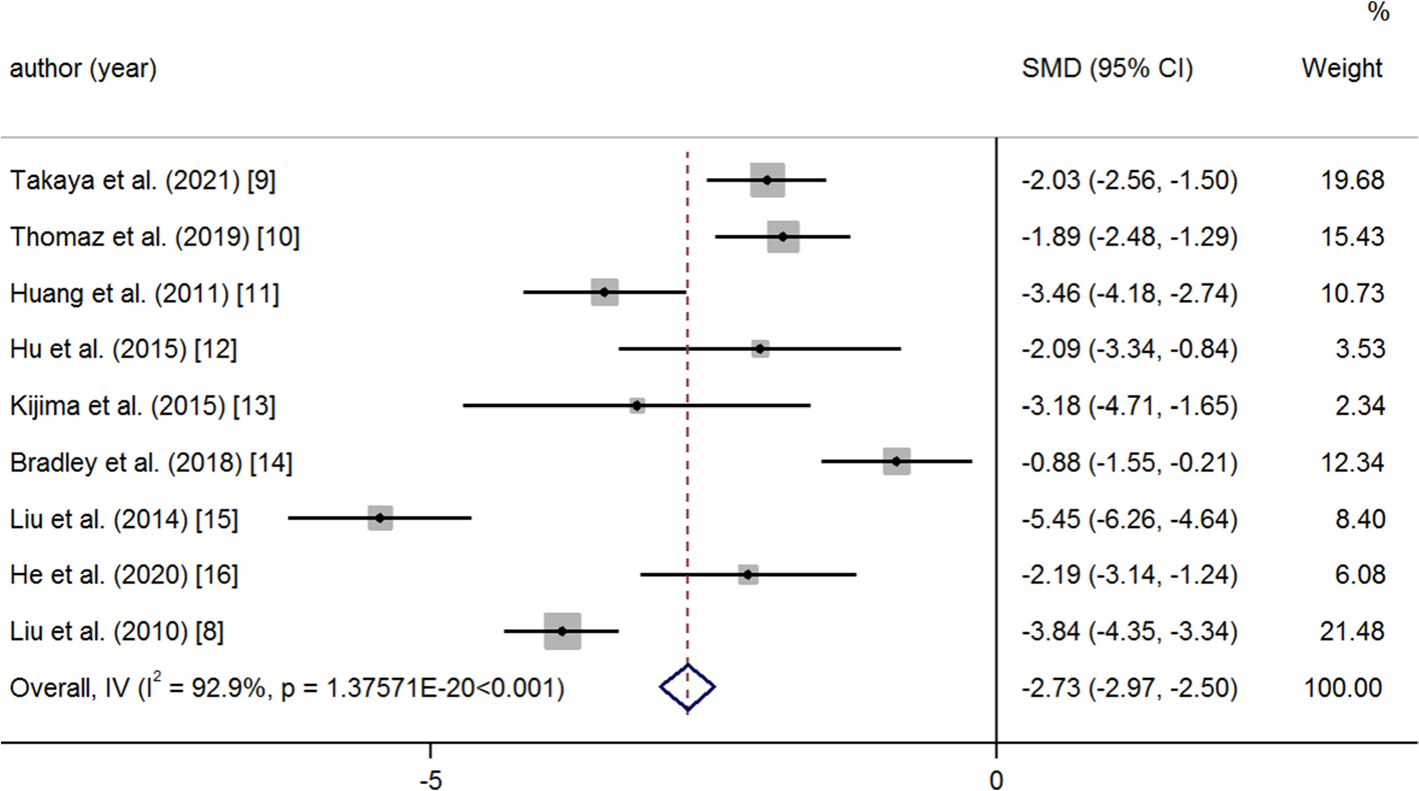
Meta-analysis of total SMDs based on the PAP

**Fig. 3:**
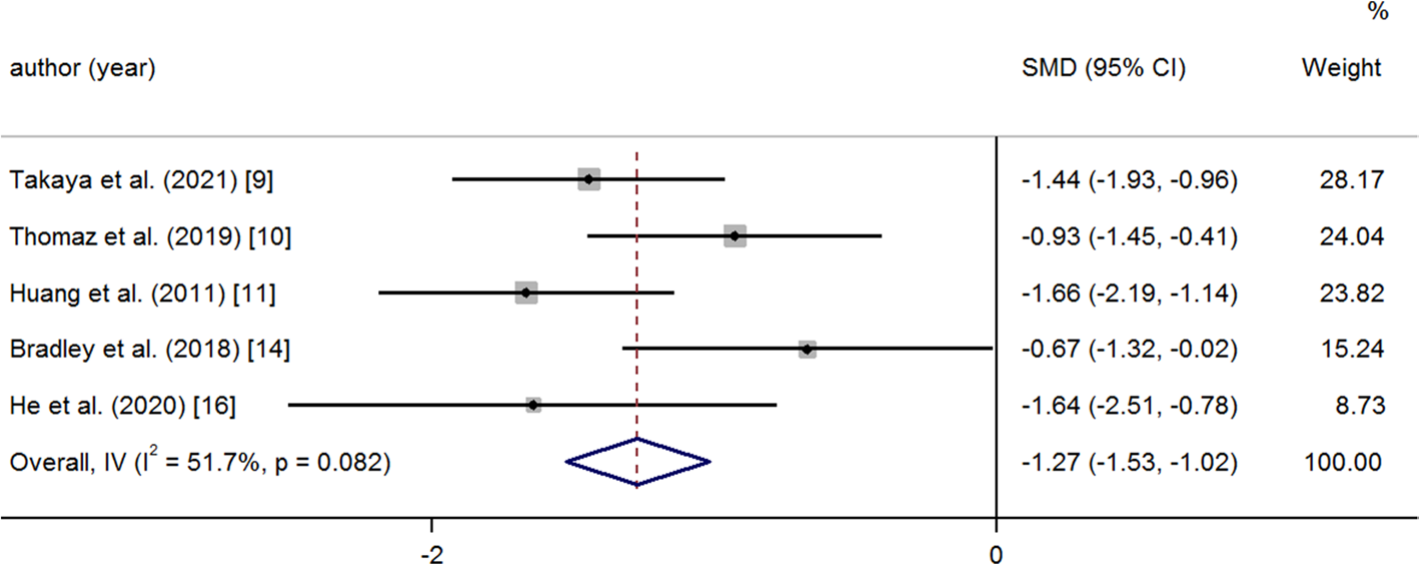
Meta-analysis of total SMDs based on the PVR

**Fig. 4:**
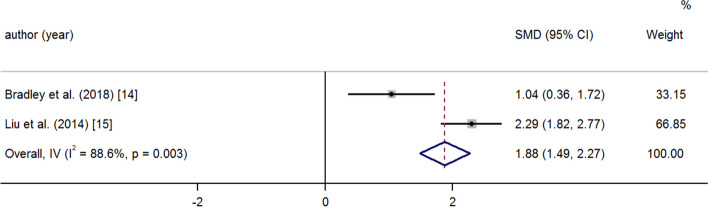
Meta-analysis of total SMDs based on the 6MWD

**Fig. 5:**
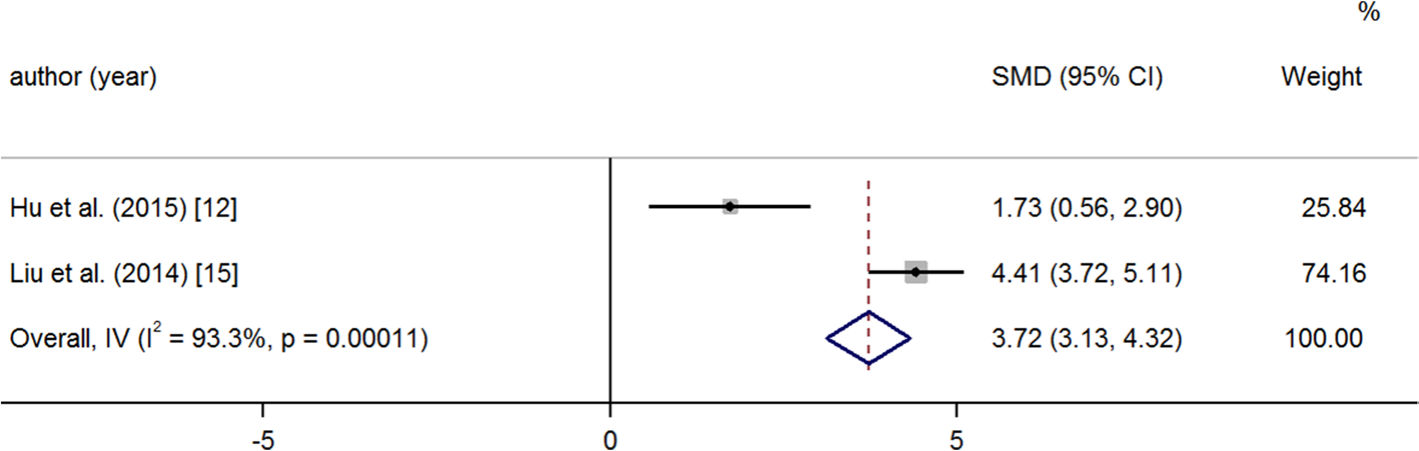
Meta-analysis of total SMDs based on SpO_2_

**Fig. 6:**
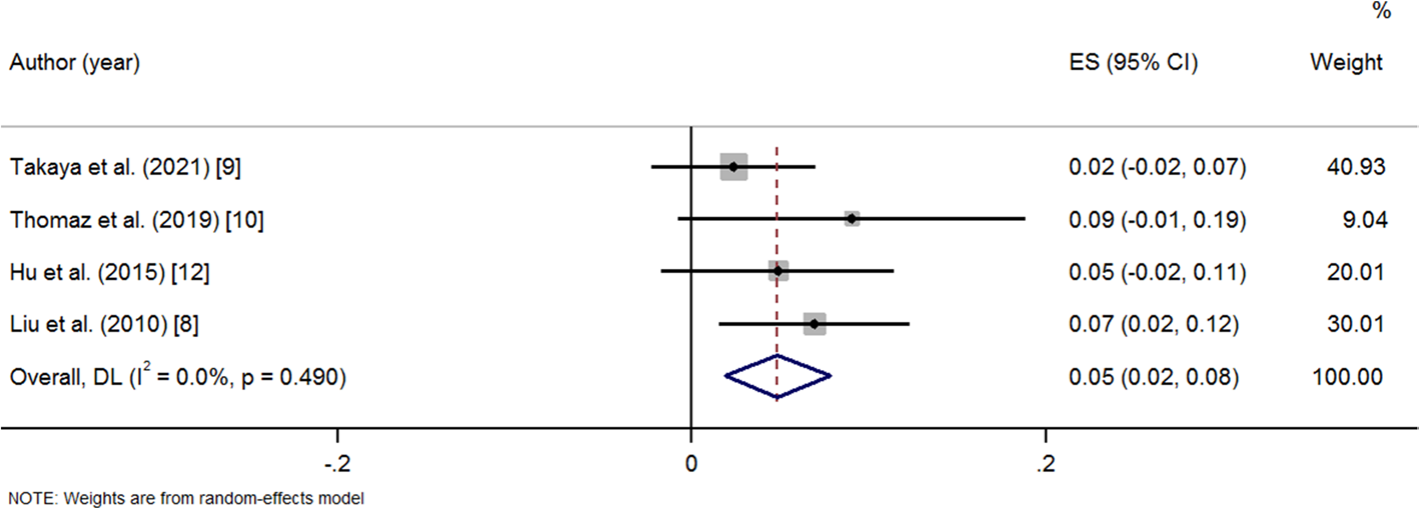
Meta-analysis of total ESs based on the mortality rate

After medication and operation, the PAP and PVR decreased significantly at the last follow-up. A random effects model was used because of great heterogeneity (PAP: SMD -2.73 95% CI -2.97, − 2.50 *p* = < 0.001 I^2^ = 92.9%) (Fig. 2); (PVR: SMD -1.27 95% CI -1.53, − 1.02 *p* = < 0.001 I^2^ = 51.7%) (Fig. 3). There was a significant increase in 6MWD and SpO_2_ at the last follow-up compared to baseline (6MWD: SMD 1.88 95% CI 1.49, 2.27 *p* = < 0.001 I^2^ = 88.6%); (Fig. 4) (SpO_2_: SMD 3.72 95% CI 3.13, 4.32 *p* = < 0.001 I^2^ = 93.3%) (Fig. 5). The combined mortality rate at follow-up was 0.05 (95% CI 0.02, 0.08 *p* = 0.01 I^2^ = 0.0%) (Fig. 6).

### Subgroup analysis

**Fig. 7:**
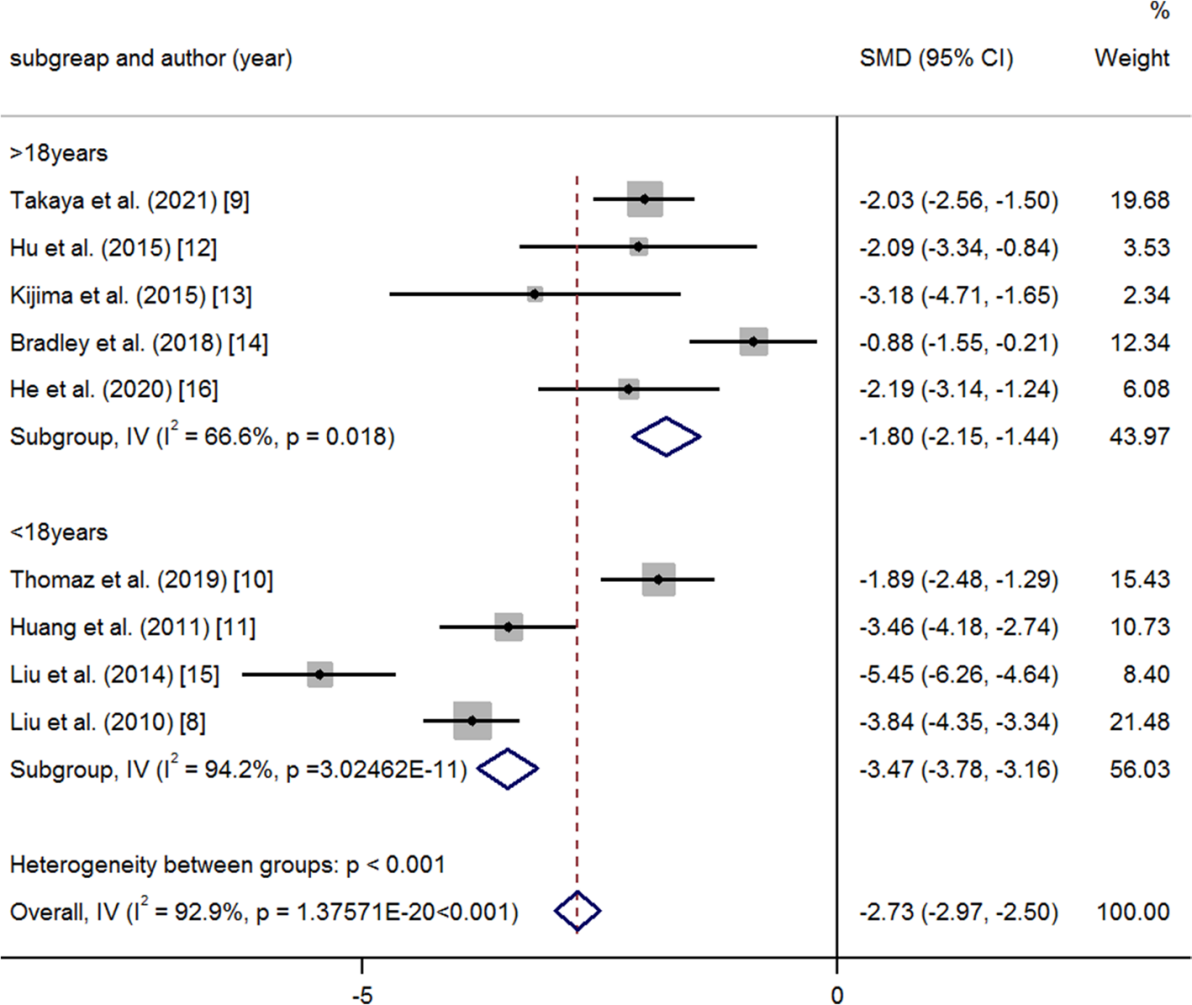
Meta-analysis of subtotal SMDs based on different ages

**Fig. 8:**
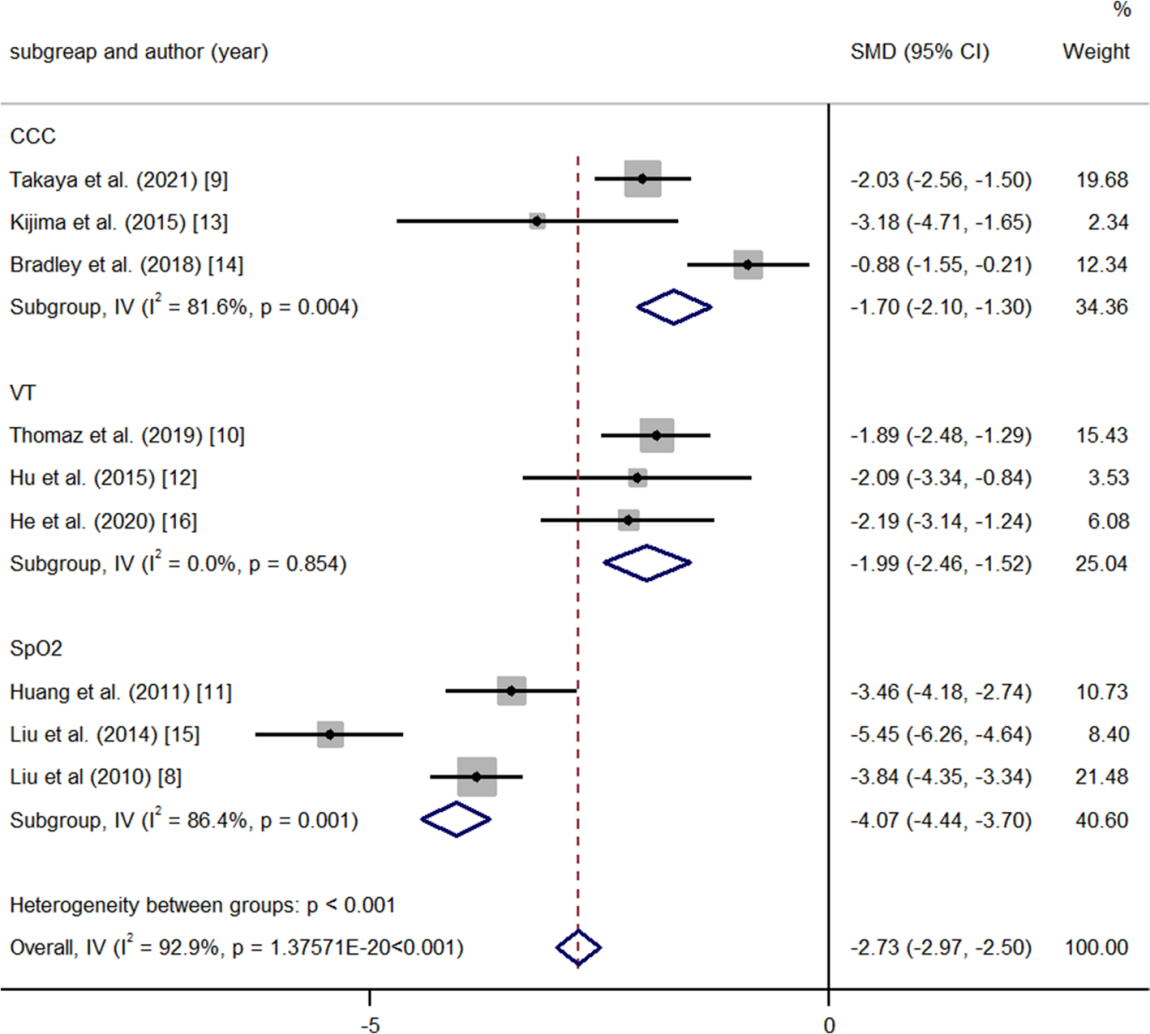
Meta-analysis of subtotal SMDs based on the different screening methods

Nine studies described the change in PAP at the time of the last follow-up. Cochrane’s Q test showed that there was significant heterogeneity in the 9 studies. According to the average age of the patients, the nine studies were divided into two groups: average age < 18 years and average age > 18 years. A random-effects model was used for subgroup analysis. The results showed that the decline in PAP was more significant in the subgroup with an average age < 18 years (> 18 years, PAP: SMD-1.80 95% CI-2.15, − 1.44, *p* = < 0.001, I^2^ = 66.6%; < 18 years, PAP: SMD-3.47 95% CI-3.78, − 3.16, *p* = < 0.001, I^2^ = 94.2%; heterogeneity between groups: *p* = < 0.001) (Fig. 7). According to the method for selecting patients, the studies were divided into the following groups: conventional cardiac catheterization (CCC), vasodilation test and (VT) and SpO_2_. The results showed that the SpO_2_ subgroup had better efficacy (CCC: PAP: SMD -1.70 95% CI -2.10, − 1.30 *p* = < 0.001 I^2^ = 81.6%; VT: PAP: SMD -1.99 95% CI -2.46, − 1.52 *p* = < 0.001 I^2^ = 0.0%; SpO_2_: PAP: SMD -4.07 95% CI -4.44, − 3.70 *p* = < 0.001 I^2^ = 86.4% heterogeneity between groups: *p* = < 0.001) (Fig. 8). Heterogeneity decreased after the subgroup analysis. Meta-regression analysis was performed, and the regression coefficient was 1.193 (95% CI: 0.269-2.118 *p* = 0.019).

### Sensitivity analysis and publication bias

**Fig. 9:**
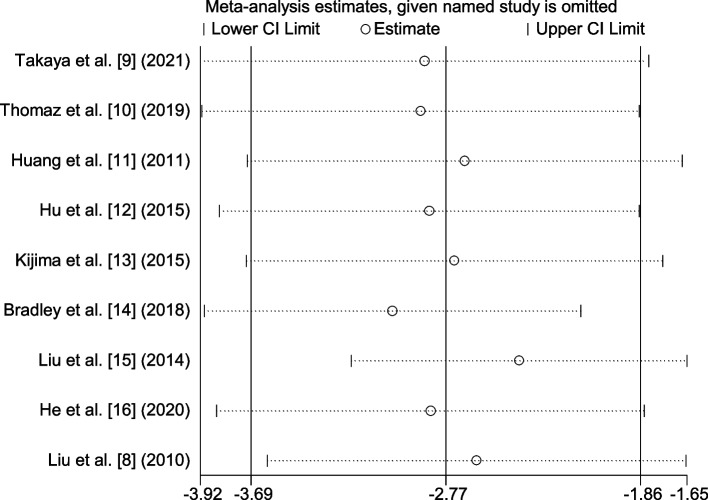
Sensitivity analysis plot

**Fig. 10:**
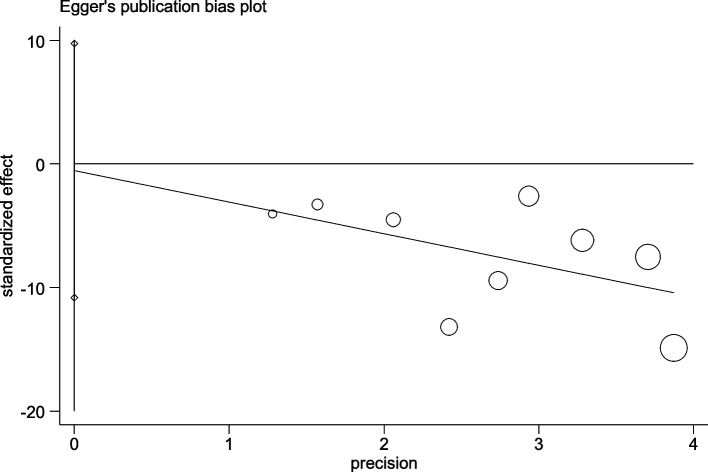
Egger test plot for PAP

For PAP, the sensitivity analysis results are shown in Fig. 9. Regardless of which study was removed, the combined effect value was still statistically significant. The Egger test model was used for analysis to assess publication bias. For PAP, the results suggested that there was no significant bias (*p* = 0.907) (Fig. 10). For PVR, *p* = 0.912. For the mortality rate, *p* = 0.296.

## Discussion

In this meta-analysis, we found that the TRT strategy could reduce the PAP and PVR and improve the 6MWD and SpO_2_ at the short-term follow-up. However, regarding PAP, there was considerable heterogeneity between studies (I^2^ = 92.9%). We observed significant differences across studies in the patient age distribution, type of CHD, targeted drug therapy, surgical screening criteria, surgical modalities and follow-up time. These may be the causes of heterogeneity. In the study of Rabinovitch et al. [25], patients younger than 2 years of age exhibited reverse remodelling in the pulmonary circulation. Age may be a factor that affects prognosis. Therefore, we conducted a subgroup analysis, and we found that the subgroup with an average age of less than 18 years had a more significant decline in PAP. We thus demonstrated that age may be associated with patient outcomes. CCC was used to screen patients. CCC is the recommended method in the guidelines and can be a good diagnostic modality for PAH [26]. The pulmonary vasodilation test and SpO_2_ were also used to screen patients. However, regardless of the screening methods, some patients still have PAH after surgery, and the proportion of patients requiring targeted drug therapy at the last follow-up varies from 43 to 100%. We designed subgroups according to different screening methods, hoping to analyse the screening method with the best efficacy. The results showed that the heterogeneity within each subgroup was lower than that within the overall combined effect. We conducted meta-regression, and the results showed that the difference in the screening methods may be the source of heterogeneity. Patients screened according to their SpO_2_ levels had a greater reduction in PAP. Spo_2_ is associated with patient prognosis. Based on previous experience, SpO_2_ has been associated with perioperative death, postoperative PAH, and adverse events such as heart failure and arrhythmia during follow-up [27–29]. Therefore, the change in SpO_2_ may be an effective screening standard to help us assess whether the patient has the opportunity for operation.

In terms of safety. Four studies found that patients had died during follow-up. The combined mortality rate was 5% at follow-up. Despite rigorous preoperative evaluations, patients still die after surgery. In the study by Thomaz et al. [10], three patients whose lung biopsies showed grades I or II according to the Heath-Edwards classification [30] died after surgery. Two of these deaths were due to pulmonary hypertension crises. Their PVRs were 3.5 Wu, 5.0 Wu and 5.2 Wu, respectively. Although right heart catheterization is the gold standard for diagnosing PAH [31], many patients still have postoperative PAH. At present, there is no effective method to accurately determine the feasibility of surgery in patients with severe PAH-CHD. Surgical indications should be carefully controlled. Risk prediction models that include age, SpO_2_, haemodynamic parameters and other relevant risk factors may better assist clinicians in decision-making.

### Study limitations

First, because of the low incidence of PAH-CHD, the number of articles and subjects included in this meta-analysis was small. As none of the 9 single-arm studies had control group, we were not able to compare long-term outcomes between the TRT strategy and simple targeted drug therapy. More randomized controlled studies are needed to confirm the long-term efficacy. Second, long-term follow-up survival data were not available in the included studies. Some patients still had residual PAH after the operation [32], and they still needed to take PAH-specific medications or even a combination of multiple drugs [33]. High medical expenses will increase the economic burden on patients and reduce their compliance. However, whether the disease will deteriorate rapidly after drug withdrawal remains to be studied. Third, some of the means and standard deviations could not be obtained directly from the included studies, and the corresponding authors could not be contacted by email. We used WebPlotDigitizer version 4.1 to obtain the medians, maximums, minimums, and interquartile values. The mean and standard deviation were obtained by calculation, which may have reduced the reliability of our results. Therefore, we are cautious about drawing conclusions.

## Conclusions

In conclusion, this meta-analysis confirms that the TRT strategy may have positive effects on haemodynamics and cardiac function in patients with severe PAH-CHD at short-term follow-up. Our analysis suggests that age and SpO_2_ changes could be potential predictors of patient prognosis. More research will be performed to build predictive models in the future.

## Data Availability

The datasets supporting the conclusions of this article are included within the article.

## Abbreviations

TRT: Treat-repair-treat
PAH-CHD: Pulmonary arterial hypertension associated with congenital heart disease
ASD: Atrial septal defect
VSD: Ventricular septal defect
PDA: Patent ductus arteriosus
A-V canal: Atrioventricular septal defect
ES: Eisenmenger syndrome
d-TGA: Dextrotransposition of the great arteries
TBA: Taussig-Bing anomaly
PAP: Pulmonary artery pressure
PVR: Pulmonary vascular resistance
Qp:Qs: Pulmonary to systemic blood flow ratio
SVR: Systemic vascular resistance
mPAP: Main outcomes: mean pulmonary artery pressure
sPAP: Systolic pulmonary artery pressure
6MWD: 6-minute walk distance
SpO_2_: Transcutaneous oxygen saturation
ERA: Endothelin receptor antagonists
PDE5i: Phosphodiesterase type-5 inhibitors
PC: Prostacyclins
sGC: Soluble guanylate cyclase stimulators
CCC: Conventional cardiac catheterization
VT: Vasodilation test
